# Violence Depicted in Superhero-Based Films Stratified by Protagonist/Antagonist and Gender

**DOI:** 10.7759/cureus.6843

**Published:** 2020-02-01

**Authors:** John N Muller, Annie Moroco, Justin Loloi, Austin Portolese, Bryan H Wakefield, Tonya S King, Robert Olympia

**Affiliations:** 1 Emergency Medicine, Penn State College of Medicine, Hershey, USA; 2 Internal Medicine, Penn State Hershey Medical Center, Hershey, USA; 3 Chemistry, Coastal Carolina University, Myrtle Beach, USA; 4 Epidemiology and Public Health, Penn State College of Medicine, Hershey, USA; 5 Emergency Medicine and Pediatrics, Penn State Hershey Medical Center, Hershey, USA

**Keywords:** violence, film, superheroes, gender

## Abstract

The objective of this study was to describe and quantify acts of violence depicted in a select number of superhero-based films, further stratified by protagonist/antagonist characters and gender. A total of 10 superhero-based films released in 2015-2016 were analyzed by five independent reviewers. The average number of acts of violence associated with protagonist and antagonist characters for all included films was 22.7 and 17.5 mean events per hour, respectively (p=0.019). The average number of acts of violence associated with male and female characters for all included films was 33.4 and 6.5 mean events per hour, respectively (p<0.001). The most common acts of violence for all major characters were “fighting”, “use of a lethal weapon”, “bullying/intimidation/torture”, “destruction of property”, and “murder” (14.9, 11.4, 3.5, 3.4, and 2.4 mean events per hour, respectively). Based on our sample of superhero-based films, acts of violence were associated more with protagonist characters and male characters.

## Introduction

Superhero-based films have become incredibly popular with both children and adults. Since 2000, there have been more than 100 films with superheroes depicted, grossing more than 23 billion dollars worldwide [1]. Despite this increase in popularity, superhero-based films represent a genre that frequently portrays violence. Published studies examining the effect of violence in the media has led the American Academy of Pediatrics to issue a policy statement, concluding that exposure to violence in the media offers a significant health risk to children that may result in aggression, bullying, antisocial attitudes, and sleep disturbances [2-6]. Furthermore, the Motion Picture Association of America’s film rating system (www.mpaa.org) does not accurately predict the frequency of violence in each rating category, and parents often find the various media rating systems difficult to use [7]. Therefore, children and adolescents may be viewing films deemed inappropriate for them based on their age.

Conversely, superheroes themselves are typically viewed as good and altruistic people that serve as role models for many children. Their origin stories often depict disadvantaged and humble beginnings, making them likable and relatable characters. In an analysis of 20 superheroes’ origin stories, 86% were orphaned or abandoned, 49% had at least one parent murdered, and 29% were bullied; this may promote resilience in vulnerable children [8]. Additional research has shown that superhero storylines may promote prosocial behavior in autistic children and encourage healthy eating habits [9-10].

In a recently published study examining positive and negative themes depicted in a selected number of superhero-based films, the authors concluded that the prevalence of negative themes, especially acts of violence, outweighed positive themes [11]. These acts of violence often included physical altercations, use of guns/knives/lethal weapons, bullying/intimidation/torture, murder, and demonstrating excessive anger. However, to the authors’ knowledge, there have been no published studies examining whether violence depicted in a superhero-based film was associated with protagonist or antagonist characters, or associated with male or female characters. Superheroes depicted in film may be viewed by children and adolescents as “the good guy”, and therefore these viewers may be influenced by their portrayal of risk-taking behaviors and acts of violence. Similarly, young girls, in particular, may be influenced by the behaviors of female superhero characters depicted in the film. The objective of this study was to describe and quantify acts of violence depicted in a selected number of superhero-based films, further stratified by protagonist/antagonist characters and gender.

## Materials and methods

We conducted a content analysis study examining acts of violence depicted in superhero-based films released during 2015 and 2016, further stratified by major protagonist/antagonist and male/female characters. Ten films included in the analysis were identified on a popular online comprehensive film database (boxofficemojo.com), limited by genre (“superhero”) and date of release (“2015” or “2016”), and chosen based on the highest lifetime gross profit as listed on July 1, 2017 (Table 1). Films were excluded if they were not super-hero based. The exclusion was not based on assigned film rating by the Motion Picture Association of American film rating system (www.mpaa.org), and thus included films assigned a PG-13 (parents strongly cautioned, some material may be inappropriate for children under 13) and R (restricted - under 17 requires accompanying parent or adult guardian) rating. 

**Table 1:** Description of the superhero-based films included in the analysis USD: United States dollar; TMNT: teenage mutant Ninja turtles; PG-13: Parents strongly cautioned, some material may be inappropriate for children under 13; R: restricted - under 17 requires accompanying parent or adult guardian

Title	Rating	Year Released	Running time (Minutes)	Lifetime Gross (million USD)	Protagonists	Antagonists
Ant-Man	PG-13	2015	118	180.2	Scott/Ant-Man; Hank Pym; Luis; Hope	Darren Cross/Yellow Jacket; Falcon
Avengers Age of Ultron	PG-13	2015	142	459.0	Captain America; Ironman; Thor; Hulk; Black Widow; Hawkeye; War Machine; Vision; Falcon; Nick Fury	Ultron & his robots; Quicksilver; Scarlet Witch; Baron Wolfgang von Strucker/Hydra
Batman the Killing Joke	R	2016	86	3.8	Batman; Batgirl; Commissioner Gordon	Joker; Paris
Batman vs Superman	PG-13	2016	183	330.4	Batman; Superman; Lois Lane; Wonder Woman	Lex Luther; Doomsday; Antoli
Captain America: Civil War	PG-13	2016	148	408.1	Avengers Team 1 (Captain America, Falcon/Sam Wilson, Ant-Man, Hawkeye, Scarlet Witch); Bucky not under mind control; Black Panther	Avengers Team 2 (Iron Man/Tony Stark, Vision, Rhodes/War Machine, Spiderman, Black Widow); Bucky under mind control (Winter Soldier); Crossbones; Zemo; Government
Deadpool	R	2016	108	363.1	Deadpool; Colossus; Negasonic Teenage Warhead	Ajax/Francis; Angel Dust
Fantastic Four	PG-13	2015	107	56.1	Reed; Johnny; Susan; Ben; Franklin Storm	Victor von Doom; Dr. Allen
Suicide Squad	PG-13	2016	137	325.1	Deadshot; Harley Quinn; El Diablo; Killer Croc; Boomerang; Slipknot; Katana; Lieutenant Edwards; Flag; Army Men	Enchantress; Waller; Incubus; Joker; Griggs; Enchantress minions
TMNT: Out of Shadows	PG-13	2016	112	82.1	Leonardo; Donatello; Raphael; Michelangelo; Splinter; April O’Neil; Vern Fenwick; Officer Casey Jones; NYPD Chief Rebecca Vincent	Shredder; Lieutenant Karai Baxter Stockman; Foot Clan; Bebop; Rocksteady; Krang
X-men: Apocalypse	PG-13	2016	147	155.4	Professor X; Mystique; Beast; Quicksilver; Cyclops; Jean; Nightcrawler; Jubilee; Moira	En Sabah Nur/Apocalypse; 4 Horsemen (Magneto, Angel, Storm, Psylocke); Government group holding Wolverine; William Stryker

Each of ten included films was viewed in its entirety by the study investigators prior to data collection. Consensus was implemented to determine which protagonist and antagonist characters played a significant role in the storyline of the film, and thus would be considered a major protagonist and antagonist character. Data analysis was performed on 66 major protagonist and 44 major antagonist characters, and 88 male and 22 female major characters. Definitions for acts of violence were created by the study investigators prior to data collection (Table 2).

**Table 2:** Definitions of acts of violence

Acts of violence	Definitions
Bullying/intimidation/torture	Unwanted aggressive behavior that involves a real or perceived power imbalance
Child maltreatment	Abuse and neglect of children under 18 years of age. It includes all types of physical and/or emotional ill-treatment, sexual abuse, neglect, negligence and commercial or other child exploitation
Demonstrating excessive anger	Demonstrating a strong feeling of displeasure, annoyance or hostility without use of physical violence
Destruction of property	Intentional damage to or the destruction of public or private property, caused by a person who is not its owner, as a result of violence
Fighting	A violent physical struggle between at least two opponents
Intimate partner violence	Behavior in an intimate relationship that causes physical, sexual or psychological harm, including physical aggression, sexual coercion, psychological abuse and controlling behaviors
Mass murder	The act of murdering a number of people (> 4), typically simultaneously or over a relatively short period of time and in close geographic proximity
Murder	The act of murdering <4 individuals
Risk taking behavior leading to harm	An impulsive action that causes harm to others or oneself
Self-directed violence	Intentional acts of hurting oneself include suicidal attempts or self-mutilation
Sexual violence	Any sexual act, attempt to obtain a sexual act, unwanted sexual comments or advances, or acts to traffic, or otherwise directed against a person’s sexuality using coercion, by any person regardless of their relationship to the victim, in any setting
Use of lethal weapons	Use of an individual weapon that is capable of causing death

A data collection instrument, developed by the study investigators, allowed the five viewers (John Muller, Annie Moroco, Justin Loloi, Austin Portolese, Bryan Wakefield) to document acts of violence performed by major protagonist and antagonist characters. Each of the five viewers watched and coded every film independently. Certain coding guidelines were decided prior to viewing the study films. For example, when coding an extended fight sequence with several major characters involved simultaneously, each contained battle involving at least two opponents was coded as one act of violence event (“fighting”), while each use of a lethal weapon (“Use of lethal weapon”) or each death (“Murder” or “Mass murder”, depending on number of deaths) was coded individually per event and per character during that given fight sequence. Acts of violence performed in the film and then later referenced were coded only at the initial encounter.

After coding, data collection instruments were collected by the primary investigator (John Muller) and the data were entered into Excel. Repeated measures Poisson regression was used to determine the overall rates of acts of violence per hour for major protagonist and antagonist characters, as well as male and female characters. These event rates were reported with corresponding 95% confidence intervals and compared between rating types with adjustment for variability among reviewers. Individual types of violence were also evaluated for protagonists, antagonists, males, and females in the same type of repeated measures Poisson regression models. The most common acts of violence were identified for each of the films separately.

The Institutional Review Board at the Pennsylvania State Hershey Medical Center deemed the study exempt.

## Results

Table 3 describes acts of violence for all included films, as well as stratified by major protagonist/antagonist and male/female (Table 3). The overall rate of acts of violence performed by protagonist characters was 22.7 (95% CI 16.8-30.7) mean events per hour. The overall rate of acts of violence performed by antagonist characters was 17.5 (95% CI 13.9-21.9) mean events per hour. With adjustment for significant reviewer variability, there was a statistically significant difference between the overall rates of acts of violence performed by protagonist vs. antagonist characters (p=0.019). The rates of both protagonist and antagonist violence were not found to significantly differ (p=0.16 and p=0.25, respectively) between the two types of film ratings.

**Table 3:** Acts of violence for all included films [reported as mean events per hour (95% confidence interval)], then stratified by protagonist/antagonist and male/female

Act of Violence	Mean Events per hour for all included films	Mean events per hour for all protagonists	Mean events per hour for all antagonists	Mean events per hour for all male characters	Mean events per hour for all female characters
Fighting	14.9 (10.9-20.2)	9.4 (6.9-13.0)	5.5 (3.8-8.1)	12.1 (8.9-16.5)	2.8 (1.9-4.0)
Use of a Lethal Weapon	11.4 (8.4-15.3)	6.0 (3.8-10.0)	5.5 (4.5-7.1)	9.6 (7.4-12.5)	1.7 (0.9-3.4)
Bullying/Intimidation/Torture	3.5 (2.5-5.0)	1.3 (0.9-2.0)	2.2 (1.6-3.1)	2.9 (2.1-3.8)	0.6 (0.3-1.6)
Destruction of Property	3.4 (2.7-4.3)	1.8 (1.5-2.3)	1.7 (1.3-2.4)	3.0 (2.4-3.9)	0.3 (0.2-0.6)
Murder	2.4 (1.6-3.7)	1.6 (0.8-2.9)	0.9 (0.6-1.1)	2.0 (1.4-3.0)	0.4 (0.2-0.9)
Mass Murder	1.8 (1.1-2.8)	0.9 (0.5-1.8)	0.8 (0.6-1.3)	1.5 (1.0-2.2)	0.2 (0.1-0.9)
Demonstrating Excessive Anger	1.4 (1.0-1.9)	1.0 (0.7-1.5)	0.4 (0.2-0.6)	1.2 (0.9-1.6)	0.2 (0.1-0.5)
Risk-Taking Behavior leading to Harm	0.9 (0.7-1.2)	0.6 (0.4-0.9)	0.2 (0.1-0.4)	0.7 (0.5-1.0)	0.2 (0.1-0.4)
Self-directed Violence	0.3 (0.1-0.6)	0.1 (0.1-0.4)	0.1 (0.03-0.4)	0.2 (0.1-0.4)	0.1 (0.01-0.4)
Sexual Violence	0.1 (0.03-0.5)	0.01 (0.001-0.06)	0.1 (0.03-0.5)	0.1 (0.03-0.5)	0
Child Maltreatment	0.1 (0.02 – 0.2)	0	0.1 (0.01-0.21)	0.1 (0.02-0.2)	0
Intimate Partner Violence	0.09 (0.02-0.5)	0.03 (0.004-0.2)	0.07 (0.01-0.3)	0.09 (0.02-0.5)	0

The frequency of “fighting” events was found to significantly differ between protagonist and antagonist characters [9.4 (95% CI 6.9-13.0) vs. 5.5 (95% CI 3.8-8.1), p<0.001]. No other acts of violence showed a statistically significant difference between protagonist and antagonist.

The overall rate of acts of violence performed by male characters significantly differed [33.4 (95% CI 26.3-40.8) mean events per hour] compared with the overall rate of acts of violence performed by female characters [6.5 (95% CI 3.7-10.9) mean events per hour], p<0.001 with adjustment for significant variability among reviewers.

Moderate to good agreement among the reviewers was found using the intraclass correlation coefficient. Among the five reviewers, agreement among the frequency of acts of violence by the protagonist was 0.73, by the antagonist was 0.60, by the males was 0.57, and by the females was 0.82. Poisson regression models did indicate significant variability among the reviewers for each of the types of acts of violence (p<0.001).

## Discussion

Based on our sample of superhero-based films, protagonist characters performed significantly more acts of violence compared to antagonist characters. This contradicts the common assumption that protagonists are the “good guys,” and therefore perform lesser acts of violence compared with their “evil” counterparts. Furthermore, we found statistically significantly more acts of violence performed by male characters compared with female characters. This discrepancy may be due to the predominance of male leading characters in superhero-based films. Over time, the number of female characters in superhero-based films appears to be increasing, with more female characters present in such films as Wonder Woman (2017) and Captain Marvel (2019). Future studies may be necessary to determine whether acts of violence performed by male and female characters differ with the increasing popularity and portrayal of female superhero and villains, potentially affecting the image adopted by pediatric viewers.

Although the Motion Picture Association of America provides a rating system to guide appropriate film viewing, this system does not accurately stratify the frequency of violent acts [7]. Our findings support the discrepancy present in the rating system, as we observed no statistically significant difference in the rate of violent acts performed by protagonist and antagonist characters between PG-13 and R-rated films. Thus, the number and type of acts of violence should be considered when applying the rating system to films.

The association between physical aggression and exposure to violent media has been previously published [12-16]. The amount of violence present in films has doubled since 1950, and gun violence present in PG-13 rated films has tripled since 1985 [17]. Children are known to learn from the observation of others’ behavior. Further, after observing that a behavior leads to a desired outcome, children often then try that behavior themselves. As superheroes are typically depicted in the media as “good”, children may view protagonist characters as role models. Therefore, children may interpret the behavior of a superhero to be acceptable, even when they are committing severely violent acts, such as “use of a lethal weapon”, “murder”, and “mass murder”. This relationship between violence depicted in the media and more frequent aggressive behavior has been found in several published studies [3, 18-20]. Furthermore, McCrary suggested that television superheroes may influence the development of moral values in kindergarten-aged children, and Martin found that the feelings children have towards superheroes are related to the way in which they feel about themselves [21-22].

Exposure to violence depicted in superhero-based films may also affect older children and adolescents. Violent acts performed by adolescent and young adults, such as physical fighting, use of a lethal weapon, mass murder, and suicide, has been prevalent in our society. In 2015, 23% of high school students in the United States reported being involved in a physical fight and 16% of high school students in the United States reported carrying a weapon [23]. A recently published study has shown that 60% of mass school shootings in the United States in the 20th century were perpetrated by adolescents, aged 11-18, and so far this century, 77 % of the mass school shootings have been carried out by adolescents [24]. Furthermore, there has been an increase in self-harm performed by children, with the rate of suicide in preteens, aged 10-14 years doubling between 2007 and 2014 [25]. It has been shown that early exposure to violence confers a risk for suicide attempt and particularly suicide death in youth [26]. While there have been no published studies examining the exposure to violence depicted in superhero-based films and violent acts performed by adolescents and young adults, future studies should focus on this potential correlation. 

The rate of bullying and cyberbullying has significantly increased over the past 10 years. In fact, according to the National Center for Education Statistics (2016), 20.8% of students in the United States reported being bullied, and of those students, 13% were made fun of, called names, or insulted; 12% were the subject of rumors; 5% were pushed, shoved, tripped, or spit on; and 5% were excluded from activities on purpose [27]. Bullying has been linked to increases in violent behavior [28]. Furthermore, an increase in exposure to antisocial media content is related to an increase in cyberbullying [29]. Based on our sample of superhero-based films, bullying/intimidation/torture is prevalent by both protagonists and antagonists, and therefore it is important to consider that children and adolescents may be learning these behaviors from the heroes and villains they see in superhero-based films.

An antidote to the increased violence depicted in superhero-based films involves co-viewing these movies as a family. Children, particularly aged 8 to 12 years, desire conversations with parents about violence. In passive co-viewing of media, there is an implicit message sent to the children that the parents approve of the content being viewed and a corresponding increase in aggressive behavior can be seen. However, if parents take an active role in their children’s media consumption via active mediation, changes are found in media-influenced behavior [30]. Active mediation occurs when parents discuss what it is being watched. This method encourages the development of critical thinking and internally regulated values. With regard to violence depicted in superhero-based films, we recommend that emphasis be placed on conflict resolution and respecting other's individuality.

There are several limitations to our study, primarily related to the selection and coding of films. We chose the 10 highest grossing superhero-based films released in 2015 and 2016 based on a popular film website. Thus, our results may not be generalizable to superhero-based films released before and since our chosen time period. Furthermore, since our selection was based on total box office gross profit, our sample of films may not represent the most popular or most watched films by children and adolescents during that time period, and pediatric viewers may access films online via streaming services that may not be reflected in the box office revenue. Lastly, we did not include any PG films in the analysis and therefore biases the results towards the more graphic violent films.

The coding of the films also represents a limitation. We found some variability in the number of events coded by each reviewer. Although coding guidelines were decided prior to our viewing of the study films, each reviewer may have interpreted scenarios, dialogue, and fighting sequences in the study films differently. Furthermore, all the reviewers were adults, who may interpret acts of violence differently than children and adolescents. Nevertheless, although our objective was to quantify acts of violence depicted in a select number of superhero-based films, the actual number of events may not be as important as the frequency in the depiction of acts of violence stratified by protagonist/antagonist and gender. Lastly, we neither did determine and quantify the intention by the major character in performing the depicted act of violence, nor did we consider the graphic nature of the violence. For example, the intention of a protagonist character in fighting or using a lethal weapon against an antagonist or causing destruction of property to protect a family or save a city would be different than the intention of an antagonist character in using a lethal weapon causing murder or mass murder, depicting massive hemorrhage, decapitations, or extremity amputations, to seek revenge against the protagonist. Although this distinction in the intention of a major character to perform an act of violence might be common sense to adolescents and adults, it may be less clear for children, and thus children may view what a superhero does as being acceptable even if the act is violent or graphic by nature.

## Conclusions

Based on our sample of superhero-based films, acts of violence were associated with protagonist characters and male characters. Therefore, pediatric health care providers should educate families on the violence depicted in this genre of film and the potential dangers that may occur when children attempt to emulate these perceived heroes. To combat the inevitable inclusion of violence in superhero-based films, the authors suggest co-viewing via active mediation, emphasizing effective communication, identification of points of agreement and disagreement, and peaceful conflict resolution in dealing with disputes or dissension instead of resorting to acts of violence.
